# A Shorter Circular Stapler Height at the Gastrojejunostomy during a Roux-En-Y Gastric Bypass Results in Less Strictures and Bleeding Complications

**DOI:** 10.1155/2018/6959786

**Published:** 2018-05-29

**Authors:** Michael Horkoff, Kieran Purich, Noah Switzer, Shalvin Prasad, Neal Church, Xinzhe Shi, Philip Mitchell, Estifanos Debru, Shahzeer Karmali, Richdeep Gill

**Affiliations:** ^1^Department of Surgery, University of Calgary, 1023 North Tower, 1403–29 Street NW, Calgary, AB, Canada T2N 2T9; ^2^Faculty of Medicine and Dentistry, 2J2.00 WC Mackenzie Health Sciences Centre, University of Alberta, 8440 112 Street NW, Edmonton, AB, Canada T6G 2R7; ^3^Department of Surgery, 2D WC Mackenzie Health Sciences Centre, University of Alberta, 8440 112 Street NW, Edmonton, AB, Canada T6G 2B7; ^4^Centre for the Advancement of Minimally Invasive Surgery (CAMIS), Royal Alexandra Hospital, 5th Floor, 10240 Kingsway Ave., Edmonton, AB, Canada T5H 3V9

## Abstract

The laparoscopic Roux-en-Y gastric bypass (LRYGB) is prone to a number of complications, most notably at the gastrojejunostomy (GJ) staple line. The circular stapler technique is a common method used to create the GJ anastomosis. Although recent studies have shown a decreased rate of anastomotic strictures with shorter stapler heights, the optimal circular stapler height to use remains controversial. We therefore completed a retrospective cohort study within the Alberta Provincial Bariatric Program (APBP) to compare outcomes between the 3.5 mm and 4.8 mm stapler heights. We identified 215 patients who had a LRYGB done between the years 2015 and 2017. 143 patients had the GJ constructed with a 3.5 mm circular stapler height, with the remaining 72 patients having the GJ fashioned with a 4.8 mm stapler height. The rate of anastomotic stricturing was lower in the 3.5 mm stapler group compared to the other cohort (3.5 versus 13.9%, resp., *p*=0.008). Likewise, the overall rate of bleeding complications was lower in the 3.5 mm stapler group compared to the 4.8 mm group (6.3 versus 15.3%, resp., *p*=0.04). The rate of anastomotic stricturing and postoperative bleeding is lower with the use of a 3.5 mm circular stapler compared to a 4.8 mm circular stapler when forming the GJ.

## 1. Introduction

Bariatric surgery is an effective intervention for obesity, resulting in significant weight loss and a decrease in obesity-related comorbidities [1]. The laparoscopic Roux-en-Y gastric bypass (LRYGB) is considered the gold-standard operative procedure for managing obesity, and the number of LRYGBs performed continues to increase worldwide [2, 3].

Although the complication rate of LRYGB is deemed to be acceptable, significant morbidity exists with anastomotic leaks, bleeding, marginal ulcer formation, and strictures [3]. The majority of these complications occur at the gastrojejunostomy (GJ) anastomosis [4, 5].

Creation of the GJ is commonly completed using a circular stapler with transoral passage of the anvil [6]. The stapler height used is thought to impact the rate of GJ-associated complications, with recent studies demonstrating a significantly lower rate of anastomotic strictures when a 3.5 mm stapler height is used compared to the traditional 4.8 mm stapler height [7–9]. More data are required to determine the effects of a shorter stapler height on rates of significant bleeding and anastomotic leaks.

The Alberta Provincial Bariatric Program (APBP) is a multicenter Canadian program made up of surgical groups at two hospitals. LRYGB is done routinely at both sites in the same technical manner. However, the two sites vary in the circular stapler height used to fashion the GJ with one site using the traditional 4.8 mm stapler and the other using a 3.5 mm stapler.

The aim of this study was to compare outcomes within the APBP between the 3.5 mm and 4.8 mm stapler heights used to fashion the GJ during LRYGB.

## 2. Methods

Patients underwent a LRYGB at two Canadian centers with the standardized surgical technique between the years 2015 and 2017. Surgeons at one center were trained with those from the other center thereby adding to the uniformity of surgical technique between sites.

Briefly, the jejunojejunostomy is created in a side-to-side manner using an Echelon 60 mm stapler (Ethicon). The biliopancreatic limb is measured to 50 cm, while the Roux limb is approximated at 100 cm. A gastric pouch is created along the lesser curve of the stomach using the Echelon 60 mm stapler and a 50-French bougie as a guide. The gastrojejunostomy is then fashioned by introducing an EEA 25 mm stapler (3.5 mm stapler height at site #1 versus 4.8 mm stapler height at site #2, (Medtronic)) through an enlarged lateral port site. The Orvil device is passed transorally. Intraoperative upper endoscopy and a leak test with insufflation across the anastomosis are performed at the surgeon's discretion.

Patients are routinely treated with low-molecular weight heparin perioperatively and are discharged home with 8 weeks of proton-pump inhibitor therapy. On postoperative day 1, all patients undergo an upper gastrointestinal contrast study to ensure anastomotic patency and integrity. Patients with significant hemodynamic changes in the postoperative period undergo an upper endoscopy and/or an exploratory laparoscopy at the surgeon's discretion.

We performed a retrospective analysis comparing outcomes between patients who had the GJ created with a 25 mm EEA stapler that had staple heights of 3.5 mm versus 4.8 mm. All patients who had surgery at site #1 had the GJ constructed with the 3.5 mm stapler height. All but one patient at site #2 had the GJ constructed with the 4.8 mm stapler height, with the other patient's GJ constructed with the 3.5 mm stapler. The follow-up time for this study was 6 months. No patients were lost to follow-up during this interval. Data were collected on preoperative factors including age, gender, body mass index (BMI), obesity-associated comorbidities (hypertension, dyslipidemia, diabetes mellitus, and obstructive sleep apnea), smoking status, preoperative anticoagulation use, and preoperative hemoglobin levels. We recorded perioperative and postoperative complication events within 6 months after surgery including hemorrhage (defined as the need for a blood transfusion prior to discharge), anastomotic stenosis requiring balloon dilation with endoscopy, marginal ulcer formation identified on upper endoscopy, anastomotic leaks, all cause reoperation and return emergency room visits, intensive care unit admission rate, and mortality.

All statistical analyses were performed using Graphpad Prism 7 software. For continuous variables, normality was first tested using the D'Agostino and Pearson normality test. Normally distributed variables were then compared using the unpaired *t*-test. Those that were not normally distributed were compared using the Mann–Whitney test. Categorical outcomes were compared using Fisher's exact test.

The appropriate institutional ethics approval was obtained for this study.

## 3. Results

Data on 215 patients who underwent a LRYGB were collected. Of those, 143 had the GJ constructed using a 3.5 mm circular stapler height, with the remaining 72 patients having the GJ constructed with the 4.8 mm circular stapler height. Patient demographics and preoperative clinical information are demonstrated in Table 1. Of note, there was no difference in preoperative age, BMI, obesity-related comorbidities, or preoperative hemoglobin between the two groups. No significant intraoperative complications were observed.

Recorded postoperative complications are depicted in Table 2. Postoperative bleeding requiring a blood transfusion was significantly lower in patients where the 3.5 mm stapler height was used compared to the 4.8 mm stapler height group (6.3% versus 15.3%, *p*=0.04). There was no difference in the transfusion threshold (hemoglobin of 91 versus 88 g/L, *p*=0.62) or the number of units of blood delivered (2.1 versus 2.9, *p*=0.24) between the groups. The source of bleeding was primarily intraluminal in the 4.8 mm group.

The occurrence of GJ anastomotic strictures requiring balloon dilation was significantly less in the 3.5 mm stapler height group compared to the 4.8 mm group (3.5% versus 13.9%, *p*=0.008). There was no difference in the average time to first dilation (61.4 days versus 55.8 days, *p*=0.79) or the total number of dilations required (2.4 versus 2.3, *p*=0.94).

There was no significant difference in the rate of marginal ulcer formation (4.9% versus 4.2%, *p*=0.99) between the 3.5 mm and 4.8 mm stapler height groups, respectively. Likewise, there was no difference in the rate of anastomotic leaks (0.7% versus 0.0%, *p*=0.99), intensive care unit admissions (0.0% versus 2.8%, *p*=0.11), or rate of reoperation (6.0% versus 9.7%, *p*=0.41). No mortalities were recorded in our cohort.

From an aggregate point of view, the 3.5 mm stapler group had a significantly shorter length of stay in hospital compared to the 4.8 mm stapler group (2.5 days versus 3.1 days, *p*=0.0001).

## 4. Discussion

The LRYGB is an effective surgical procedure for the long-term management of obesity [1]. However, complications resulting in significant patient morbidity do occur. Anastomotic bleeding, stricturing, marginal ulcer formation, and leaks are all possible, particularly at the gastrojejunostomy site [3]. The optimal circular stapler height to minimize these GJ complications continues to be debated.

In this study, we retrospectively compared the outcomes of patients who underwent a standardized LRYGB within the APBP using either a 4.8 mm or 3.5 mm circular stapler height to fashion the GJ. We identified a significantly lower rate of hemorrhage and a lower rate of anastomotic stricturing at the GJ with the use of a 3.5 mm stapler height when compared to the 4.8 mm height.

The rational for using a shorter stapler height to reduce the risk of anastomotic bleeding pertains to the greater tissue compression that is provided. Optimal tissue compression, where adequate hemostasis is achieved without creating significant ischemia or tissue shearing, is dependent on intrinsic tissue thickness, elasticity, and robustness of blood supply [10]. To date, there remain to be little published data on various device-tissue interactions and specifically how intrinsic tissue factors change when stapled [11]. In general, the incidence of intraluminal bleeding after LRYGB is reported at 1.9% to 4.4% with a trend towards a reduced incidence when using a shorter 3.5 mm stapler height that results in presumably more tissue compression [7–9, 12]. Our study supports the use of a 3.5 mm stapler height to reduce post-LRYGB hemorrhage compared to a 4.8 mm stapler height. We recognize that our reported rates of bleeding requiring a blood transfusion are higher than the literature; however, the transfusion threshold within the APBP is quite liberal and may account for the discrepancy. Importantly, we did not appreciate an increase in anastomotic leaks when using the 3.5 mm stapler height in our cohort.

Our work is in concordance with others that have previously demonstrated a similar reduction in the rate of anastomotic strictures with a 3.5 mm stapler height to between 3.9 and 6.1% [7–9]. In general, the etiology of strictures is probably related to local ischemia, small anastomotic leaks, and inflammation from local ulcerations [13, 14]. The mechanisms relating the lower rate of anastomotic strictures to stapler height are unknown but are likely multifactorial.

This study was completed as a pilot project to determine whether future comparisons between stapler heights would be warranted to improve outcomes. This work has the inherent limitations of a retrospective comparison. Also, the source of postoperative bleeding was difficult to determine in patients who were managed conservatively. Given the standardization of the operative technique, however, we are confident that the differences in bleeding rates between our groups can be attributed to the stapler height used at the GJ. Surgical experience between the two sites involved in this study is also variable and contributes to the limitations of this study. A randomized prospective trial may be best suited to add further information regarding the optimal circular stapler height for fashioning the GJ.

## 5. Conclusion

Our results suggest that use of a 3.5 mm circular stapler height compared to a 4.8 mm stapler height to fashion the GJ during LRYGB may reduce the rate of significant anastomotic bleeding and stricture formation.

## Figures and Tables

**Table 1:** Patient demographics.

Variable	3.5 mm stapler (*n*=143)	4.8 mm stapler (*n*=72)	*p* value
Age	46 ± 11	44 ± 9	0.10
Gender (female)	117 (81)	63 (89)	0.24
Body mass index (kg/m^2^)	45.8 ± 7	45.2 ± 6	0.58
Hypertension	81 (56)	31 (43)	0.06
Dyslipidemia	43 (30)	23 (31)	0.88
Diabetes mellitus	54 (38)	25 (34)	0.76
Obstructive sleep apnea	70 (49)	29 (40)	0.24
Active smoker	0 (0)	1 (1)	0.33
Preoperative anticoagulation use	3 (2)	2 (3)	0.99
Preoperative hemoglobin (g/L)	140 ± 12	139 ± 12	0.57

Data are presented as numbers with percentages in parentheses or mean ± standard deviation. ^∗^Statistically significant (*p* less than 0.05); mm, millimeter; kg/m^2^, kilograms per meter squared; g/L, grams per liter.

**Table 2:** Complications.

Variable	3.5 mm stapler (*n*=143)	4.8 mm stapler (*n*=72)	*p* value
Length of stay (days)	2.5 ± 1.0	3.1 ± 1.2	0.0001^∗^
Hemorrhage			
Requiring transfusion	9 (6.3)	11 (15.3)	0.04^∗^
Hemoglobin prior to transfusion (g/L)	91 ± 18	88 ± 12	0.62
Units of blood delivered	2.1 ± 0.9	2.9 ± 1.5	0.24
Postoperative day 1 hemoglobin (g/L)	123 ± 13	120 ± 14	0.12
Intraluminal bleeding	0	8	
Extraluminal bleeding	0	1	
Unknown source	9	2	
Anastomotic stenosis			
Number requiring dilation	5 (3.5)	10 (13.9)	0.008^∗^
Time to dilation (days)	61.4 ± 36.0	55.8 ± 34.0	0.79
Average number of dilations required	2.4 ± 1.7	2.3 ± 1.6	0.94
Marginal ulcers			
Number	7 (4.9)	3 (4.2)	0.99
Time to presentation (days)	101 ± 60	62 ± 37	0.56
Number requiring surgical revision	1 (0.7)	0 (0)	0.99
Anastomotic leaks	1 (0.7)	0 (0)	0.99
ICU admissions	0 (0)	2 (2.8)	0.11
Reoperation rate (all cause)	9 (6.0)	7 (9.7)	0.41
Re-presentation rate (all cause)	45 (31.5)	16 (22.2)	0.20
Mortality	0 (0)	0 (0)	0.99

Data are presented as numbers with percentages in parentheses or mean ± standard deviation. ^∗^Statistically significant (*p* less than 0.05); mm, millimeter; g/L, grams per liter.
